# Green Synthesis of Carbon Dots Derived from Walnut Oil and an Investigation of Their Cytotoxic and Apoptogenic Activities toward Cancer Cells

**DOI:** 10.15171/apb.2018.018

**Published:** 2018-03-18

**Authors:** Elham Arkan, Ali Barati, Mohsen Rahmanpanah, Leila Hosseinzadeh, Samaneh Moradi, Marziyeh Hajialyani

**Affiliations:** ^1^Nano Drug Delivery Research Center, Kermanshah University of Medical Sciences, Kermanshah, Iran.; ^2^Pharmaceutical Sciences Research Center, Faculty of Pharmacy, Kermanshah University of Medical Sciences, Kermanshah, Iran.

**Keywords:** Apoptogenic Activity, Carbon Quantum Dots, Human Carcinoma Cell Lines, Walnut Oil

## Abstract

***Purpose:*** This paper introduces a green and simple hydrothermal synthesis to prepare carbon quantum dots (CQDs) from walnut oil with a high quantum yield. In addition, cytotoxic and apoptogenic properties of the CQDs were analyzed on human cancer cell lines.

***Methods:*** The optical properties and morphological characteristic were investigated by the TEM, XRD, FT-IR, UV-vis and photoluminescence (PL).The cytotoxic potential of walnut CQDs was evaluated on PC3, MCF-7 and HT-29 human carcinoma cell lines using the MTT methods. The mechanism of action was studied by investigating the mode of cell death using the activation of caspase-3 and 9 as well as mitochondrial membrane potential (MMP). Cellular uptake of the CQDs was detected by fluorescence microscope. CQDs had an average size of 12 nm and a significant emission at 420 nm at an excitation wavelength of 350 nm was recorded.

***Results:*** The prepared CQDs possessed a good fluorescent quantum yield of 14.5% with quinine sulfate (quantum yield 54%) as a reference and excellent photo as well as pH stabilities. The walnut CQDs were proved to be an extremely potent cytotoxic agent, especially against MCF-7 and PC-3 cell lines. Induction of apoptosis by CQDs was accompanied by an increase in the activation of caspase-3. Caspase-9 activity did not increase after exposure to the CQDs. Additionally; the MMP did not show any significant loss.

***Conclusion:*** The results of our study can corroborate the cytotoxic and apoptotic effect of walnut CQDs in the PC3 and MCF-7 cancer cell lines.

## Introduction

Carbon-based materials have some properties such as good flexibility, high strength and stability and excellent electrical and thermal conductivity. Carbon fibers, fullerene, porous materials, carbon nanotubes and carbon quantum dots are some of the members in the carbon-based family.^1^ Carbon quantum dots (CQDs) are taken into account as a new class of nano-carbonaceous materials with photoluminescence properties discovered by Xu *et al*. (2004).^2,3^

It now seems that priority of the CQDs rather than semiconductor quantum dots and organic dyes is due to their high optical and chemical stability and biocompatibility‏.In addition, they have low metabolic degradation, bright fluorescence, low toxicity, suitable water solubility and low photo-degradation.^4,5^ These advantages suggest that the CQDs can be used as a non-toxic replacement for semiconductor quantum dots. Laser irradiation,^6^ ultrasonic treatment,^7^ hydrothermal treatment,^8^ and electrochemical oxidation,^9^ are methods developed for the preparation of the CQDs. Some of these methods have some defects such as complicated process, expensive material and equipment required and low reported yields. However, using a cost-effective and high-yield method for large scale fluorescent CQDs preparation is important. Hydrothermal method is one of the proper approaches for the production of carbon dots that can produce them from different sources of carbon including organ molecules and carbohydrates‏.^10^

Furthermore, the most important advantages of the CQDs is that they have many hydroxyl and carboxyl groups on their surface.^11^ The mentioned groups can be beneficial for therapeutic agents conjugation and biological effects in the fields of multicolor bio-labeling and bio-imaging,^12^ and catalysis.^13^Moreover, there have been several studies about anticancer effects of the CQDs.^14^ Natural products are the useful sources which could be used in the preparation of CQDs.^15^ Chi-Lin Li *et al*. used Ginger for production of CQDs and observed that the obtained CQDs selectively inhibited the growth of the HepG2 cells.^14^ In addition, the suppressor activity of green tea-derived CQDs was shown against two human breast carcinoma cell lines.^16^

The present study aims to produce CQDs by a facile, green, and low-cost hydrothermal method using walnut (*Jungleregia*) oil, as the precursor. It must be noted that there are several investigations on the anti-proliferative effects of the different parts (leaf, seed, root and green husk) of this medicinal plant.^17,18^ Next, the cytotoxic effects of the CQDs were investigated on three human cancer cell lines: PC3, MCF-7 and HT-29 cells. Moreover, the molecular mechanisms, in which CQDs exert the cytotoxic effect, were also assessed on the most sensitive cell lines.

## Materials and Methods

Hydrazine and ammonium bromide were purchased from Merck (Germany). 3-(4,5-dimethylthiazol-2yl)-2,5-diphenyltetrazoliumbromide (MTT), rhodamine, caspase 9 substrate and Caspase-3 Detection assay Kit were purchased from Sigma Aldrich (St Louis, MO, USA). Cell culture medium, penicillin–streptomycin, and fetal bovine serum (FBS) were obtained from Gibco (Gibco, Grand Island, NY, USA).

### Synthesis of Carbon Dots

In the present study, CQDs were produced using hydrothermal methods as follows.

The oil was separated from walnut and passed through Whatman filter paper to eliminate large particles. The oil was then centrifuged for 15 min at 6000 rpm for five times. Then, 30 ml of the clear walnut oil was transferred into a 100 mL Teflon-lined stainless steel laboratory autoclave and heated in an oven at temperature of 220°C for 24 h, and cooled to room temperature. The brownish solution was centrifuged at 14000 rpm for 15 min for three times to remove large or agglomerated particles and the supernatant containing CQDs was further purified using a 0.2 µm membrane.

### Quantum yield

The relative fluorescence quantum yield of resultant CQDs were evaluated at an excitation wavelength of 320 nm and the following equation was used‏:

$$Q_{x}=Q_{r}\left(\frac{I_{x}}{I_{r}}\right)\left(\frac{A_{r}}{A_{x}}\right)\left(\frac{\eta_{x}^{2}}{\eta_{r}^{2}}\right)$$

Where, r and x reflect the standard reference and the sample of interest respectively, Q is the quantum yield, I is the integrated emission spectra, η is the refractive index of the solvent, and A is the absorbance. In order to avoid self-absorption effect, the absorbance was kept below 0.1 quinine sulfate in 0.1 M H2SO4 because lead to avoid self-absorption effects. Quinine sulfate had known quantum yield of 0.54 that was selected as standard reference‏.

### Cell culture

PC3, human prostate cancer cell line, was obtained from Pasteur Institute (Tehran, Iran). The PC3 was established in 1979 from bone metastasis of grade IV of prostate cancer in a 62-year-old Caucasian male. This cell line is useful to evaluate prostatic cancer cells response to anti- cancer drugs.^19^ MCF-7 human breast cancer cells are used widely for research on chemotherapy agents for breast cancer.^20^ HT-29 is a human colorectal adenocarcinoma cell line with epithelial morphology. This cell line was established in 1964 from the primary tumor of a 44-year-old Caucasian female with colorectal adenocarcinoma.^21^ The cells were cultured in Dulbecco’s modified Eagle’s medium (DMEM-F12) with 5% (v/v) fetal bovine serum, 100 U/mL penicillin, and 100 mg/ mL streptomycin. The medium was changed 2-3 days and was sub-cultured when the cell population density reached to 70–80% confluence‏.

### Assay of inhibitory effects of CQDs on the growth of human carcinoma cell lines

The inhibitory effects of walnut CQDs on the growth of PC3, MCF-7, and HT-29 cells were evaluated by MTT assay. Briefly, the cells were suspended in a mixture of DMEM-F12 and 10% bovine serum, 100 units/mL of penicillin and 100 µg/mL of streptomycin at a concentration of 1 × 10^5^ cells/mL. The cell suspension was pipetted into a 96-well plate (100 µL /well) and was permitted to adhere in a humidified incubator containing 5% CO2 at 37 °C. 24 hours after seeding, the cells were treated with different concentrations (0-10 µg/mL) of CQDs dissolved in DMSO. After 24 h, the medium was replaced by 100 𝜇L of 0.5 mg/mL of MTT in growth medium and were incubated at 37°C for 3 hrs. Next, the supernatants were removed carefully and DMSO (100 µL) was added to each well to dissolve formazan crystals. Then, the absorbance of each well at 570 nm was determined using an Elisa plate reader (Synergy-2 of BioTek Instruments Inc., Winooski, VT, USA). For each compound the IC_50_ value was calculated by plotting the log 10 of the viability percentage versus concentration‏.

### Cellular uptake of CQDs

The cellular uptake of CQDs was evaluated in MCF-7 and PC-3 cells. Briefly, the cells were seeded in 12-well plates at a density of 5.0 × 10^5^ cells/well. After 24 h the cells were treated with the fresh medium containing the IC_50_ concentration of CQDs and followed by incubation for 4 h at 37°C in a 5% CO_2_/95% air atmosphere. Fluorescence images were taken at 100 magnifications under a fluorescence microscope (Micros AUSTR1A) with imaging system. The cells without any CQDs treatment were used as a comparative control.

### Assessment of Mitochondrial Membrane Potential (MMP)

Rhodamine 123 florescent dye, a cell permeable cationic dye, was used in MMP assay. Depolarization of MMP during cell apoptosis results in the loss of rhodamine 123 from the mitochondria and a decrease in intracellular florescence ntensity.^22^ At the end of treatment, cells were incubated with rhodamine 123 for 30 min at 37 °C. The fluorescence intensity was measured at an excitation wavelength of 488 nm and an emission wavelength of 520 nm using a florescence microplate reader (BioTek, H1M, USA)‏.

### Measurement of caspase-3 and caspase-9 activities

The caspases activities were determined based on the manual of the sigma Caspase-3 assay kit. Briefly, cells were detached and lysed with lysis buffer containing protease inhibitors. The lysed cells centrifuged for 10 min at 14000 rpm. Next, the supernatant was transferred to a tube and mixed with caspase-3 and caspase-9 substrates. After 1 h incubation at 37°C, the absorbance of the chromophore p-nitroanilide was detected by a microplate reader at 405 nm. As a control, non-treated cells were analyzed and the data were expressed by percentage of control‏.

### Statistical analysis

All data were analyzed using one-way ANOVA using Graph Pad Prism software (GraphPad software, San Diego, CA, USA). Differences between the mean± SEM (standard error of the mean) of samples were considered significant at P < 0.05. The IC_50_ values were generated from the MTT results using GraphPad Prism software.

## Results and Discussion

### Characterization of CQDs

**Figure 1:**
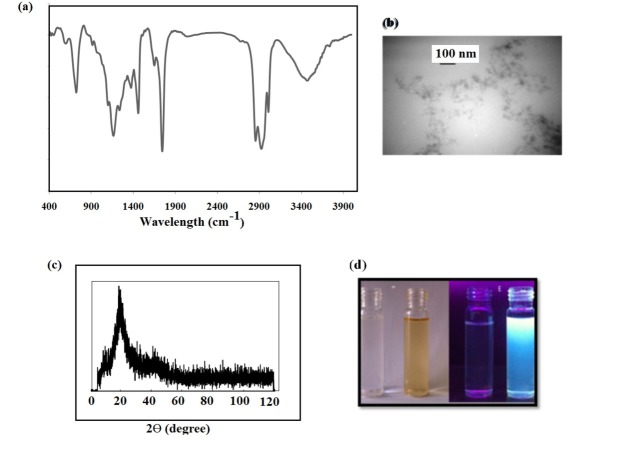
(a) FTIR spectra, (b) TEM images at different magnifications 100 nm of CQDs and (c) XRD pattern of CQDs prepared from walnut oil. The photographs of prepared CQDs in solution under visible light (d) and 365 nm UV lamp light (e).

In the FTIR spectra of the CQDs, the spread band observed at 3200-3500 cm^-1^ belonged to C–OH and N–H stretching vibrations. Also, one at 2800-2950 cm^-1^ was assigned to the C–H stretching vibrations (Figure 1-a). The bending vibrations of the N–H could appear at 1400 cm^-1^. Peaks appearing at approximately 1600 and 1280 cm^-1^ indicated the presence of C=O and C–NH–C stretching vibration, respectively. The band at approximately 1066 cm^-1^ presented the existence of C–O (hydroxyl, ester, epoxide or ether) groups.

The TEM images (Figure 1-b) showed that the CQDs were spherical as well as monodisperse and had a narrow size distribution. The average diameter of the CQDs was 12.3 ± 2.7 nm.

The XRD pattern of the prepared CQDs (Figure 1-c) displayed a broad peak centered at approximately 2θ=20°, indicating a graphitic nature with highly disordered carbon atoms.

The elementary composition of the prepared CQDs was confirmed by elemental analysis. The results for the presented atoms were: C 37.26 wt.%; N 2.25 wt.%; H 4.07 wt.%; S 0.97 wt.% and O (calculated) 55.45 wt.%. We also showed the diluted walnut oil and walnut CQDs in hexane under room light (Figure 1-d) and UV light (365 nm) (Figure 1-e).

### Optical Properties of the CQDs

The optical properties of the CQDs were investigated by photoluminescence (PL) and excitation spectra of the prepared CQDs at room temperature.

**Figure 2:**
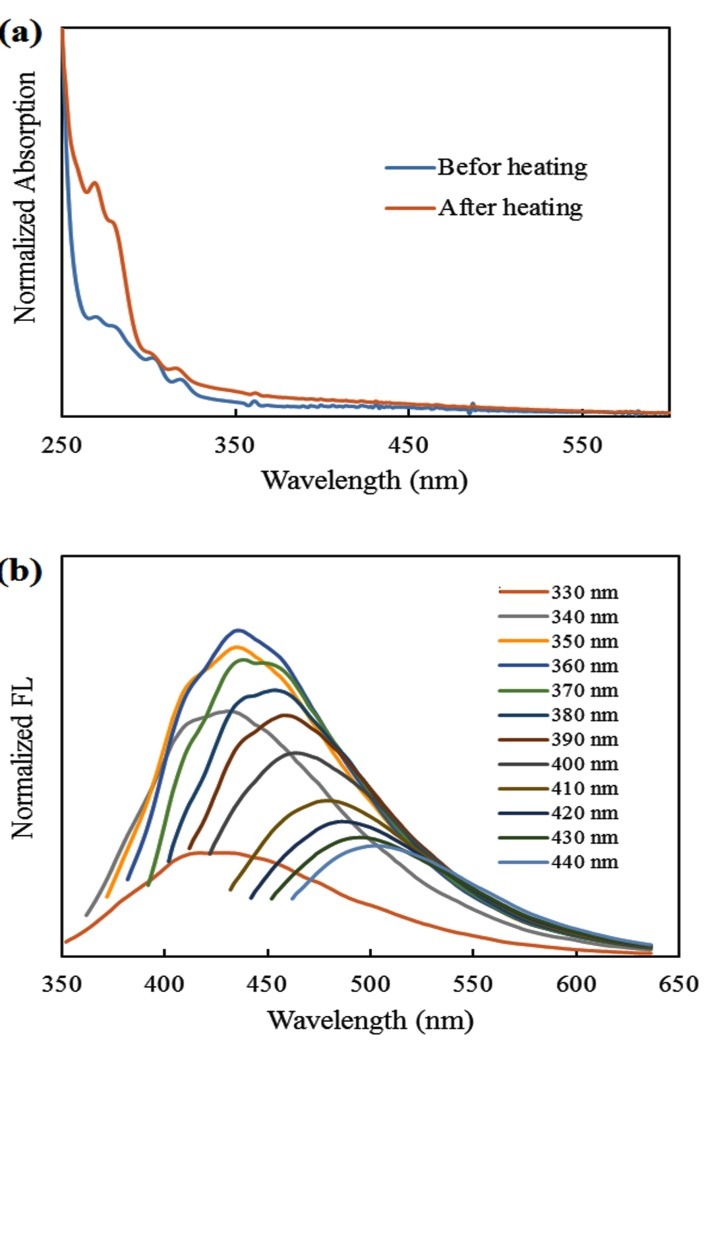
(a) UV–Vis absorption spectrum before and after converting the walnut oil to CQDs (b) UV-vis absorption and PL emission spectra of the prepared CQDs from walnut oil.

In the UV–Vis spectrum corresponding to the CQDs, a sharp peak at220 nm and a broad peak at 270 nm (Figure 2-a) were shown. The observed peaks could be assigned to π–π* and n–π* transitions of C=C and C=O bonds. Increasing the peak at 270 nm conformed the addition of more C=O bonds to the CQD structures. The PL recorded at the excitation wavelengths ranging from 320 to 440 nm indicated a generic excitation-dependent property (Figure 2-b). The maximum PL appeared at the excitation of 360 nm with the maximum at 430 nm. The quantum yield of the CQDs was estimated to be 14.5 at an excitation wavelength of 340 nm and in the presence of quinine sulfate as a standard reference. The results showed a strong and stable PL, which was found excitation-dependent.

### Inhibition of Cell Viability 

**Figure3:**
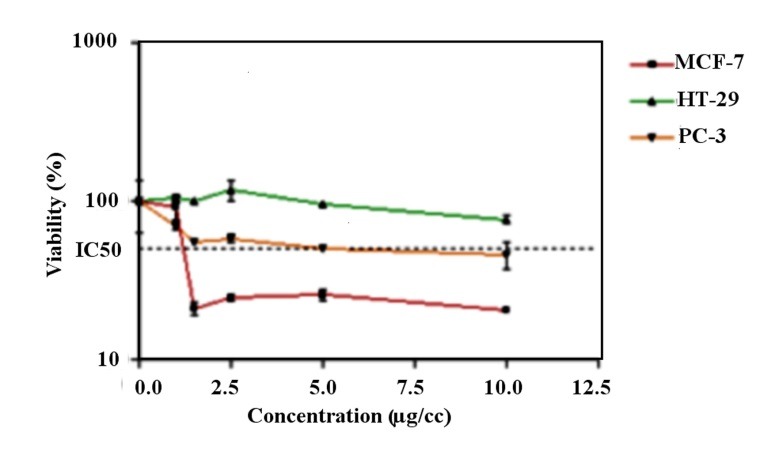
Cytotoxic effects of CQDs in human carcinoma cell lines. PC-3, MCF-7 and HT29 cells were incubated with different concentrations of CQDs for 24 h. The cell proliferation inhibition was determined by MTT assay as described under materials and methods. Data are presented as mean ± S.E.M (n=3)

MTT assay was performed in order to examine the possible anti-proliferative effect of CQDs on PC3, MCF7 and HT-29 cell lines. Complete dose-response curves were generated and IC_50_ values were calculated against three human carcinoma cell lines. Walnut CQDs have proved to be an outstandingly potent cytotoxic agent, especially against PC3 and MCF7 cell lines as confirmed by its IC_50_ value. As shown in Figure 3, exposure to the CQDs for 24 hrs resulted in a concentration-dependent decrease in cell viability, with the approximate IC_50_ of 1.25 ± 0.062 µg/cc, 5 ± 1.03µg/cc , and >10 µg/cc in MCF-7, PC-3 and human carcinoma cell lines, respectively.

The synthesized CQDs decreased the cell proliferation by 50% in the MCF-7 and PC-3 cancer cells at the concentrations of 1.25µg/mL and 5µg/mL, respectively. These values are below20 μg/mL, indicating that the CQDs potentially present an interesting cytotoxic activity toward the PC3 and MCF-7, and human carcinoma cell lines.^23,24^

As mentioned before, the different parts (leaf, seed, root and green husk) of walnut (*Jungle regia*) have shown cytotoxic effect against human carcinoma cell line. Wei *et al*. showed the inhibitory effect of the *J. regia* leaf extract on the growth of PC-3 cells (IC_50_= 48.4 μg/mL) through apoptosis. Furthermore, the cell cycle phase distribution altered after exposure to the mentioned extract in the PC-3 cell line.^2,5^ In the another study, Carvalho *et al*. evaluated the anti-proliferative effect of walnut leaf, green husk and seed methanolic extracts on renal carcinoma cell lines, A498 and 769P as well as Caco-2, human epithelial colorectal adenocarcinoma cells. Their results revealed that walnut extracts exert the slight inhibitory effect on the growth of cells.^26^

### Cellular Uptake of CQDs 

**Figure 4:**
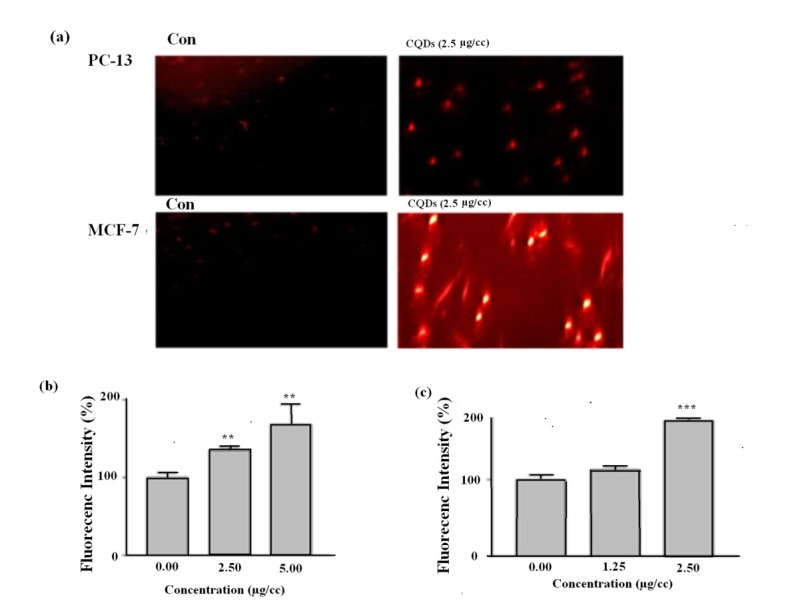
(a) Fluoroescence microscope images demonstrating the intracellular distribution in PC3 and MCF-7 cell lines.CQDs accumulation in (b) PC3 and (c) MCF-7 cells after 4 h exposure to different concentrations of CQDs. Data are presented as mean ± S.E.M (n=3). **𝑃< 0.01, ***𝑃< 0.001 versus control.

In an attempt to assess whether walnut CQDs are able to enter the cells, we performed fluorescence microscopy imaging of the cells incubated with the CQDs (Figure 4). As shown in Figure 4-a, after the treatment, we observed bright fluorescence intensity spread all over the cells incubated with the CQDs. In addition, as Figures 4-b and 4-c show, the intracellular fluorescence of the CQDs increased the dose dependently after exposure to different concentrations of the CQDs in PC3 and MCF-7 cell line, respectively.

### The apoptotic potentials of the CQDs: Effects of CQDs on Caspases – 3 and 9 activities and MMP 

**Figure 5:**
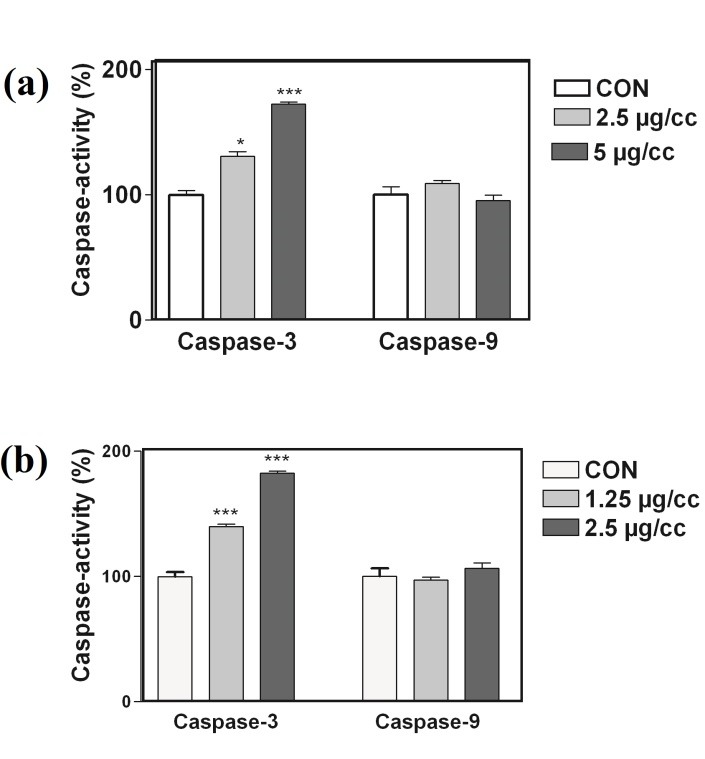
Involvement of activation of caspases in the induction of apoptosis by walnut CQDs in PC3 and MCF-7 cancer cells. Cells were incubated with CQDs for 24 h and cell lysates were assayed using microplate reader for activation caspases. Significant differences were compared with the control. Data are presented as mean ± S.E.M. ∗𝑃< 0.05,∗∗𝑃< 0.01, versus control.

Apoptosis has been accepted as a preferable mode of action of the antitumor drug,^27^ and considerable effort is directed toward the development of potential medicines inducing apoptosis in the malignant cells. Therefore, we investigated the apoptotic potentials of the CQDs on the most sensitive cell lines using some apoptosis- related parameters. Activation of caspases is well-known to play an essential role in the initiation and progression of programmed cell death. From this family, caspase-3 is an executioner caspase that proteolytically cleave many proteins playing a central role in apoptotic cell death.^28^ It serves as a target for different signaling pathways of the programmed cell death. In order to indicate the type of cell death involved in our experiments, the activity of caspase-3 and caspase-9 was examined in PC-3 and MCF-7 cells. The obtained results showed the dose-dependent alteration of the caspase-3 activity and exposure to the CQDs caused increasing the caspase-3 activity of both MCF-7 and PC3 cell lines (Figures 5-a, 5-b). The drugs used in chemotherapy, induce apoptosis through death receptor pathway (extrinsic) or at the mitochondria by stimulating the intrinsic pathway.^29^ Thepermeabilization of the mitochondrial membrane during mitochondria dependent pathway, causes bioenergetics failure and permits the release of a small hem protein, cytochrome c (Cyt c), to the cytosol, leading to caspase-9 activation.^28^ To determine which apoptotic pathway is activated by the CQDs, the activation of caspase-9 was examined. As Figure 4-b shows, 24h treatment with the CQDs was not able to increase the activation of caspase- 9 in the MCF-7 and PC-3 cell lines.

**Figure 6:**
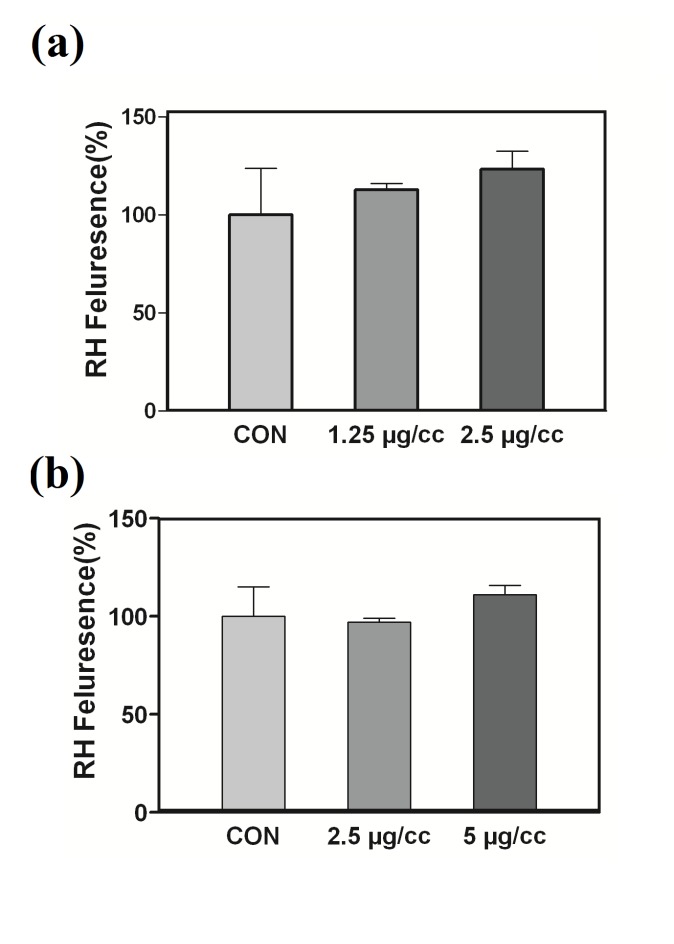
Effect of CQDs on MMP collapse.(a) MCF-7 and (b) PC3 cells were treated with different concentrations of CQDs for 24 h. Graphs show the change in MMP as represented by the mean florescence intensity (MFI) of rhodamine 123.

The involvement of the mitochondrial pathway in the CQD-mediated apoptotic cell death was also examined by measuring the MMP in the PC-3 and MCF-7 cells. As shown in Figure 5, when cells were treated with the CQDs for 24 h at 37°C, no decrease in the retention of rhodamine 123 was observed at the used concentrations (Figure 6). These results suggest that the mitochondrial pathway probably does not cause a substantial role in the CQD-induced apoptosis. In the recent study, a novel bio-peptide isolated from walnut residual protein and its anti-proliferative ability was evaluated against human carcinoma cell line. Results indicated that the isolated bio-peptide was able to inhibit cancer cells growth selectively through inducing apoptosis and could be used for cancer treatment.^30^

## Conclusion

It can be concluded that walnut carbon quantum dots have cytotoxic and apoptotic potential on prostate and breast cancer cells. Therefore, these CQDs may be considered an economical and easily accessible source of effective agents used in cancer chemotherapy.

## Acknowledgments

The authors gratefully acknowledge the research council of Kermanshah University of Medical Sciences for the financial support. This work was performed in partial fulfillment of the requirement for pharm.D of Mohsen Rahmanpanah, in faculty of pharmacy, Kermanshah University of Medical Sciences, Kermanshah, Iran.

## Ethical Issues

Not applicable.

## Conflict of Interest

The authors declare no conflict of interests.
